# Mesoscopic Constitutive Model for Predicting Failure of Bulk Metallic Glass Composites Based on the Free-Volume Model

**DOI:** 10.3390/ma11020327

**Published:** 2018-02-24

**Authors:** Yunpeng Jiang

**Affiliations:** State Key Laboratory of Mechanics and Control of Mechanical Structures, Nanjing University of Aeronautics and Astronautics, Nanjing 210016, China; ypjiang@nuaa.edu.cn; Tel.: +86-25-84892152

**Keywords:** bulk metallic glass matrix composites (BMGCs), meso-mechanics, shear band, stress–strain relation, failure

## Abstract

A meso-mechanical damage model is developed to predict the tensile damage behaviors of bulk metallic glass composites (BMGCs) toughened by ductile particles. In this model, the deformation behaviors of the BMG matrix and particles are described by the free volume model and Ludwik flow equation, respectively. Weng’s dual-phase method is used to establish the relationship between the constituents and the composite system. The strain-based Weibull probability distribution function and percolation theory are adopted in characterizing the evolution of shear bands leading to the progressive failure of BMGCs. Moreover, the present model is performed under strain-controlled loading. Comparing to experiments on various BMGCs, the predictions are in good agreement with the measured results, which confirms that the present model successfully depicts the composite properties, such as yield strength, uniform deformation and strain softening elongation.

## 1. Introduction

To improve the poor damage tolerance of pure bulk metallic glass (BMG), many kinds of composite (BMGC) systems have been prepared, and many important conclusions were reached. However, an in-depth understanding of the inherent synergic effect among different constituents in BMGCs is still lacking. In comparison to simulations and experiments, theoretical models are more efficient and convenient in explaining their micro-deformations and composite effect. Moreover, quantitative relations are more efficient in optimizing the ductility/toughness of BMGs via rapidly tuning their microstructures. It is imperative to understand the correlations among processing, microstructures and properties for such composites. According to the thermodynamics and free energy principle, Marandi et al. [1] advanced an elastic-viscoplastic constitutive model for describing the finite deformation behaviors of BMGCs. They [2] further extended their model to better predict the stress–strain relations of in-situ BMGCs. Qiao et al. [3] were the first to consider the work-hardening ability of dendrite phase and softening of metallic glass matrix from the point of micromechanics view, and the predictions are in a fairly good agreement with tensile experiments. It should be noted that the interaction among the constituents is not fully reflected. Yang et al. [4] also established an analytical model to describe the deformation kinematics, free volume evolution, hardening, softening and viscosity of BMGs. Recently, Sun et al. [5] improved Qiao’s previous micromechanics model to predict the tensile behaviors of in-situ BMGCs more accurately based on the measured data via nano-indentation. Rao et al. [6] proposed a new meso-mechanical constitutive model to predict the monotonic tensile/compressive deformation of BMGCs with toughening phases, but their analytical model has very complicated formulas. Jiang et al. [7,8,9,10] regarded the shear bands as micro-cracks, established their equivalence relation, and finally developed two micromechanics models based on the incremental tangent stiffness and secant modulus, respectively. 

These analytical models can successfully reflect some main features, such as yield strength, strain hardening and stress softening elongation, of ductile particles filled BMGs. However, they cannot fully take account of the inherent microstructure evolution and deformation features of BMG matrix. It is expected that shear bands will gradually transform into micro-cracks with the deformation increasing, and correspondingly the stress–strain curve of BMGCs will enter the stress-softening stage. To the author’s knowledge, the damage effect in the BMGCs was not addressed in these analytical models, and a simple micromechanics model is always required to describe their intriguing mechanical response.

This paper aims to build an analytical damage model for predicting the tensile failure of BMGCs toughened by ductile particles. The deformation behaviors of BMG matrix and particles are described by the free volume model and Ludwik flow equation, respectively, and Weng’s homogenization frame is adopted to establish the interaction between the constituents and composites. As compared to the other models, the present model is more convenient to apply, and more readily to be expanded. The developed model is performed under strain-controlled loading, and verified by modeling the monotonic stress–strain relations of particle toughened BMGCs.

## 2. Analytical Model of BMGCs

The BMGCs are filled with ductile particles, and the stress–strain relations of the constituents should be described with the proper constitutive equations. For such dual-phase composites, where both phases can undergo plastic flow, Weng [11] developed an analytical model to depict the stress–strain relations of the composites, and later extended by Zhu [12]. Their formula will be used as the basis of a new micromechanics method for BMGC, and a perfect interfacial bonding between two phases is assumed. For a dual-phase composite, the particle phase is referred as phase 1 and the BMG matrix as phase 0, and those of the composite are expressed by symbols without any script. All the tensors and vectors are written in boldface letters. The volume fractions for the particle and matrix phases are denoted by *c*_1_ and *c*_0_, respectively, and should satisfy the condition *c*_1_ + *c*_0_ = 1. 

### 2.1. Constitutive Model of BMGs

The shear band evolution controls the fundamental deformation mechanisms in BMGs. At the microscopic level, shear band formation is accompanying with the evolution of the local structural order. One atomistic mechanism capturing shear band formation and evolution in BMGs is the free volume model proposed by Spaepen [13] and further extended by Steif [14]. From the continuum mechanics point of view, the shear band is regarded as a consequence of strain softening and acts as a strain-localization phenomenon. This model considers free volume as an internal state variable to characterize the structural evolution of BMGs at the atomic level.

Following a *J*_2_-type, small strain visco-plasticity framework, the free volume model is adapted into the multi-axial stress state. The total strain rate in the BMG matrix is written as

(1) $${\dot{\varepsilon }}_{ij}={\dot{\varepsilon }}_{ij}^{e}+{\dot{\varepsilon }}_{ij}^{p},$$

which includes the elastic part, ${\dot{\varepsilon }}_{ij}^{e}=\frac{1+\nu }{E}\left({\dot{\sigma }}_{ij}-\frac{\nu }{1+\nu }{\dot{\sigma }}_{kk}\delta_{ij}\right)$, and the plastic part, ${\dot{\varepsilon }}_{ij}^{p}$. For the BMG matrix, the plastic strain rate, i.e., the flow equation is expressed as

(2) $${\dot{\varepsilon }}_{ij}^{p}=f\sigma_{ij}^{\prime }/\sigma_{eq},$$

where $\sigma_{ij}^{\prime }=\sigma_{ij}-\sigma_{kk}\delta_{ij}/3$ is the deviatoric stress tensor and $\sigma_{eq}={\left(\sigma_{ij}^{\prime }\sigma_{ij}^{\prime }\right)}^{1/2}$ is the von Mises′ stress. *f* is the flow stress, which is defined by

(3) $$f=f_{0}\exp\left(-\frac{\Delta G^{m}}{k_{B}T}\right)\exp\left(-\frac{1}{\xi }\right)\sinh\left(\frac{\sigma_{eq}\Omega }{2k_{B}T}\right),$$

where *f*_0_ is the frequency of atomic vibration; Δ*G^m^* is the activation energy; *k_B_* is the Boltzmann constant; *T* is the absolute temperature; Ω is the atomic volume; and *ξ* is the concentration of free volume. The free volume evolution equation under multi-axial stress state is written as

(4) $$\dot{\xi }=\frac{1}{\alpha_{0}}f_{0}\exp\left(-\frac{\Delta G^{m}}{k_{B}T}\right)\exp\left(-\frac{1}{\xi }\right)\left\{\frac{2k_{B}T}{\xi \nu^{*}S}\left(\cosh\left(\frac{\sigma_{eq}\Omega }{2k_{B}T}\right)-1\right)-\frac{1}{n_{D}}\right\},$$

where *α*_0_ is a geometrical factor of order unity; ν^*^ is a critical volume; *S* is the Eshelby modulus, given by *S* = 2(1 + *v*)*μ*/3(1 − *v*); *v* is Poisson’s ratio; *μ* is the shear modulus; and *n_D_* is the number of atomic jumps needed to annihilate a free volume equal to ν^*^ and is usually taken to be 3–10.

### 2.2. Constitutive Model of Ductile Phases

The Ludwik equation is adopted for ductile particles in terms of von Mises′ effective stress and plastic strain as

(5) $$\sigma_{eq}=\sigma_{y}+h{\left(\varepsilon_{eq}^{p}\right)}^{n},$$

where $\varepsilon_{eq}^{p}={\left(2\varepsilon_{ij}^{p}\varepsilon_{ij}^{p}/3\right)}^{1/2}$; *σ_y_*, *h* and *n* are the initial yield stress, strength coefficient and the work-hardening exponent, respectively; and these material parameters will be determined by fitting with a measured stress–strain curve. Moreover, Hencky’s flow rule is adopted,

(6) $$\varepsilon_{ij}^{p}=\frac{3}{2}\frac{\varepsilon_{eq}^{p}}{\sigma_{eq}}\sigma_{ij}^{\prime },$$

### 2.3. Homogenization Method for BMGCs

For dual-phase composites, Weng’s model is used to establish the relationship among ductile particles, matrix and the resulting composites under monotonic uniaxial tension. The detailed derivations are found in their original work [11]. The relationship between the hydrostatic and deviatoric strains of BMGCs are defined by

(7) $${\bar{\sigma }}_{kk}=3\kappa_{0}\left[1+\frac{c_{1}(\kappa_{1}-\kappa_{0})}{c_{0}\alpha_{0}^{s}(\kappa_{1}-\kappa_{0})+\kappa_{0}}\right]{\bar{\varepsilon }}_{kk},$$

(8) $${\bar{\sigma }}_{ij}^{\prime }=2\mu_{0}^{s}\left\{\left[1+\frac{c_{1}(\mu_{1}-\mu_{0}^{s})}{c_{0}\beta_{0}^{s}(\mu_{1}-\mu_{0}^{s})+\mu_{0}^{s}}\right]{\bar{\varepsilon }}_{ij}^{\prime }-\frac{c_{1}\mu_{1}}{c_{0}\beta_{0}^{s}(\mu_{1}-\mu_{0}^{s})+\mu_{0}^{s}}\varepsilon_{ij}^{p(1)}\right\},$$

where $\alpha_{0}^{s}$ and $\beta_{0}^{s}$ are the components of the classical Eshelby’s tensor for spherical inclusions, and given as

(9) $$\alpha_{0}^{s}=\frac{1+\nu_{0}^{s}}{3(1-\nu_{0}^{s})},\beta_{0}^{s}=\frac{2(4-5\nu_{0}^{s})}{15(1-\nu_{0}^{s})},$$

and *κ* and *μ* denote the bulk and shear moduli, and are written as follows to satisfy the isotropic relations,

(10) $$\kappa_{r}=\frac{E_{r}}{3(1-2v_{r})},\mu_{r}^{s}=\frac{E_{r}^{s}}{2(1+v_{r}^{s})},(r=0\;\mathrm{or}\;1)$$

where *E* and *ν* are the Young’s modulus and Poisson’s ratio, respectively; and *E* and *ν* with superscript “*s*” denote the secant modulus and secant Poisson’s ratio, respectively, defined by

(11) $$E_{r}^{s}=\frac{1}{\frac{1}{E_{r}}+\frac{\varepsilon_{eq}^{p}}{\sigma_{eq}}},v_{r}^{s}=\frac{1}{2}-\left(\frac{1}{2}-\nu_{r}\right)\frac{E_{r}^{s}}{E_{r}},(r=0\;\mathrm{or}\;1)$$

The relationship between the hydrostatic and deviatoric strains of the constituents and those of BMGC are given as

(12) $$\varepsilon_{kk}^{\left(0\right)}=\frac{\alpha_{0}^{s}(\kappa_{1}-\kappa_{0})+\kappa_{0}}{c_{0}\alpha_{0}^{s}(\kappa_{1}-\kappa_{0})+\kappa_{0}}{\bar{\varepsilon }}_{kk},$$

(13) $${\varepsilon_{ij}^{\left(0\right)}}^{\prime }=\frac{\beta_{0}^{s}(\mu_{1}-\mu_{0}^{s})+\mu_{0}^{s}}{c_{0}\beta_{0}^{s}(\mu_{1}-\mu_{0}^{s})+\mu_{0}^{s}}{\bar{\varepsilon }}_{ij}^{\prime }-c_{1}\beta_{0}^{s}\frac{\mu_{1}}{c_{0}\beta_{0}^{s}(\mu_{1}-\mu_{0}^{s})+\mu_{0}^{s}}\varepsilon_{ij}^{p(1)},$$

(14) $$\varepsilon_{kk}^{\left(1\right)}=\frac{\kappa_{0}}{c_{0}\alpha_{0}^{s}(\kappa_{1}-\kappa_{0})+\kappa_{0}}{\bar{\varepsilon }}_{kk},$$

(15) $${\varepsilon_{ij}^{\left(1\right)}}^{\prime }=\frac{\mu_{0}^{s}}{c_{0}\beta_{0}^{s}(\mu_{1}-\mu_{0}^{s})+\mu_{0}^{s}}{\bar{\varepsilon }}_{ij}^{\prime }+\frac{c_{0}\beta_{0}^{s}\mu_{1}}{c_{0}\beta_{0}^{s}(\mu_{1}-\mu_{0}^{s})+\mu_{0}^{s}}\varepsilon_{ij}^{p(1)}.$$

### 2.4. Failure of the BMG Matrix

During the deformation of BMGCs, shear bands gradually transform into micro-cracks with the applied loading, and therefore are simplified as micro-cracks. Moreover, shear bands lead to the stress softening behavior, which is similar to the effect of micro-cracks on the mechanical behaviors [15]. From this viewpoint, shear bands are equivalent to micro-cracks, and then some analytical models for micro-cracks are also applied to the strain localization effect induced by shear bands [16].

The representative volume element (RVE) is often utilized to account for the micro-crack orientation statistics in the inhomogeneous materials. The micro-cracks generated by shear bands are supposed to be random, and thus the corresponding effective moduli are given by [17]

(16) $$\frac{E}{E_{in}}={\left[1+\frac{16(1-\nu_{in}^{2})(1-3\nu_{in}/10)}{9(1-\nu_{in}/2)}\rho \right]}^{-1},$$

(17) $$\frac{G}{G_{in}}={\left[1+\frac{16(1-\nu_{in})(1-\nu_{in}/5)}{9(1-\nu_{in}/2)}\rho \right]}^{-1},$$

(18) $$\frac{\nu }{\nu_{in}}=\frac{E}{E_{in}}\left[1+\frac{8(1-\nu_{in}^{2})}{45(1-\nu_{in}/2)}\rho \right],$$

where the subscript “*in*” denotes the intact materials with no micro-cracks. For the ductile phases, the failure criterion based on statistical probability is associated with strain levels. The strain-based Weibull distribution function is introduced to characterize the shear band induced fracture as

(19) $$P(\varepsilon_{p})=1-\exp\left[-{\left(\frac{\varepsilon_{p}}{\varepsilon_{0}}\right)}^{m}\right],$$

where *ε_p_* is the plastic strain and *ε*_0_ is the reference strain, and since there is no data available for parameter *m*, which is need to be determined by fitting from a final stage with damage so that the predicted stress–strain relations can duplicate the experiments. Then, the density of shear-bands in the BMGCs is defined by

(20) $$\rho =\rho_{0}\cdot P(\varepsilon_{p})=\rho_{0}\cdot \left\{1-\exp\left[-{\left(\frac{\varepsilon_{p}}{\varepsilon_{0}}\right)}^{m}\right]\right\},$$

where *ρ*_0_ denotes the saturate density of shear-bands. After introducing the percolation threshold of shear-band propagation in the BMG matrix, the shear-band density is given by

(21) $$\rho =\left\{ \begin{array}{l} \rho_{0}\cdot P(\varepsilon_{p})(1-c_{1})\;(c_{1}>c_{cr})\\ \rho_{0}\cdot P(\varepsilon_{p})(1-c_{1})\left[1-{\left(c_{cr}-c_{1}\right)}^{\chi }\right]\;(c_{1}<c_{cr}) \end{array}\right.,$$

where *c_cr_* and *χ* are constants, which will be assigned by the experimental data. In fact, almost the precipitates are randomly distributed in real BMGCs. Therefore, some necessary statistics experiments should be performed to examine the flaw sensitivity and reliability of BMGs.

## 3. Numerical Implementation

The developed model is performed under strain-controlled loading, and the detailed algorithm is explained here. For a time interval from *t_n_* to *t_n_*_+1_ (*∆t_n_*_+1_ = *t_n_*_+1_ − *t_n_*), the necessary variables at time *t_n_*, such as ${\bar{\sigma }}_{n}$, ${\bar{\varepsilon }}_{n}$, $\varepsilon_{n}^{\left(0\right)}$, $\varepsilon_{n}^{\left(1\right)}$, $\sigma_{n}^{\left(0\right)}$ and $\sigma_{n}^{\left(1\right)}$, are known, and a uniform strain increment $\Delta {\bar{\varepsilon }}_{n+1}$ is given. The average strain increments $\Delta \varepsilon_{n+1}^{\left(r\right)}\;(r=0,\;1)$ in each phase are determined by Equations (12)–(15), and then the secant modulus, secant Poisson′s ratio and plastic strain can be computed by the constitutive models for each phase. The overall stress increment corresponding to the current strain increment can be solved.

The key issue in the modeling procedure is to fix $\Delta \varepsilon_{n+1}^{\left(r\right)}\;(r=0,\;1)$. At first, the initial tentative value of $\Delta \varepsilon_{n+1}^{\left(1\right)}$ is given by

(22) $$\Delta \varepsilon_{n+1}^{\left(1\right)}\;=\Delta {\bar{\varepsilon }}_{n+1}.$$

Then,

(23) $$\Delta \varepsilon_{n+1}^{\left(0\right)}\;=\frac{\Delta {\bar{\varepsilon }}_{n+1}-c_{1}\Delta \varepsilon_{n+1}^{\left(1\right)}}{1-c_{1}}.$$

Then, the compatibility of a strain increment $\Delta \varepsilon_{n+1}^{\left(1\right)}$ in particles is checked by the residual ***R*** as follows

(24) $$\begin{array}{ll} R_{ij} & =\frac{\kappa_{0}}{f_{0}\alpha_{0}^{s}(\kappa_{1}-\kappa_{0})+\kappa_{0}}{\bar{\varepsilon }}_{kk}^{n+1}+\frac{\mu_{0}^{s}}{f_{0}\beta_{0}^{s}(\mu_{1}-\mu_{0}^{s})+\mu_{0}^{s}}{\bar{\varepsilon }}_{\prime ij}^{n+1}\\ & +f_{0}\beta_{0}^{s}\frac{\mu_{1}}{f_{0}\beta_{0}^{s}(\mu_{1}-\mu_{0}^{s})+\mu_{0}^{s}}{\varepsilon_{ij}^{p(1)}|}_{n+1}-\varepsilon_{n}^{\left(1\right)}-\Delta \varepsilon_{n+1}^{\left(1\right)} \end{array},$$

***R*** represents the difference between particle′s tentative average strain increment and that obtained by Weng′s model. If ‖R‖ < TOL (**R**→**0**), the iteration stops. Otherwise, $\Delta \varepsilon_{n+1}^{\left(1\right)}$ is updated with another new iteration,

(25) $$\Delta \varepsilon_{n+1}^{\left(1\right)}=\Delta \varepsilon_{n+1}^{\left(1\right)}+R.$$

In the uni-axial strain-controlled loading, only the strain increment in loading direction ($\Delta {\bar{\varepsilon }}_{11}$) is exactly given, and the others should be determined by the overall-stress constraints. An additional iteration procedure should be performed to obtain the values of $\Delta {\bar{\varepsilon }}_{22}$ ($\Delta {\bar{\varepsilon }}_{33}$) as

(26) $${\bar{\sigma }}_{22}={\bar{\sigma }}_{33}=0.$$

For a given strain increment ($\Delta {\bar{\varepsilon }}_{11},\;\Delta {\bar{\varepsilon }}_{22}=\Delta {\bar{\varepsilon }}_{33}$), determining the exact value of $\Delta {\bar{\varepsilon }}_{22}$ should be a key procedure in the computation. A simple method is explained here. The iteration of updating $\Delta {\bar{\varepsilon }}_{22}$ is also performed from an initial value of zero, and a new $\Delta {\bar{\varepsilon }}_{22}$ = $\Delta {\bar{\varepsilon }}_{22}$ + *∆* is assigned with a very small *∆*. The value of *∆* is adjusted correspondingly based on the computation precision. The iteration stops if Equation (26) is satisfied; otherwise, $\Delta {\bar{\varepsilon }}_{22}$ is updated according to the above equation for the next iteration. 

**Figure 1:**
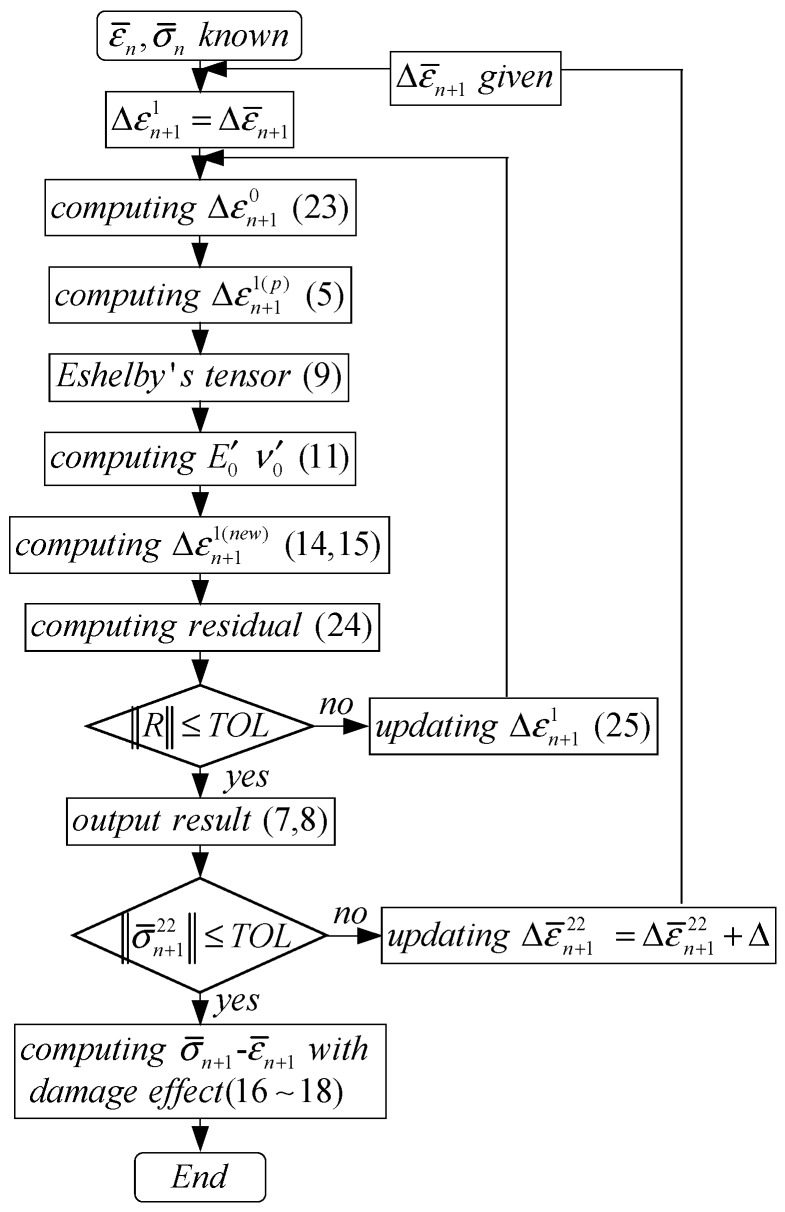
Flow chart of numerical integration algorithm under displacement-based loading.

Based on the completed stress–strain curves by the above iteration, the shear-band density *ρ* is determined, and then the overall elasticity is given. Based on Equations (16)–(18), the stress–strain curves for the BMGCs can be re-evaluated by involving the damage effect. The above-mentioned numerical implementation procedure is illustrated by the flowchart in Figure 1, and a Fortran code was programmed to predict the stress–strain relations of BMGCs.

## 4. Results and Discussion

### 4.1. Comparisons with the Experiments

**Figure 2:**
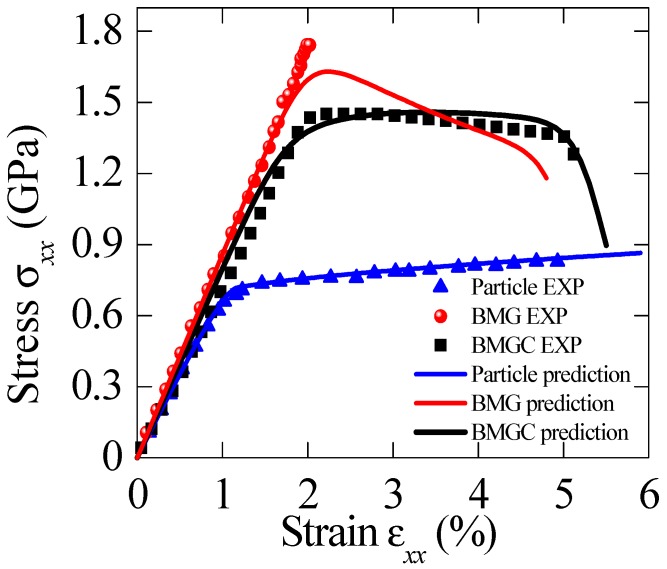
Comparison between the predictions and experiments for the BMGCs with dendrite volume fraction of *c*_1_ = 20% [18]. *ε*_0_ = 0.04 and *m* = 18 are adopted in the computation.

Szuecs et al. [18] prepared a Zr-based BMGCs with dendrite volume fraction of *c*_1_ = 20%, and measured their mechanical properties under tension. The dendrites are described by Equation (5), and their properties are: *E*_1_ = 72 Gpa, *v*_1_ = 0.4, *σ_y_* = 700 Mpa, *h* = 1200 Mpa, and *n* = 0.65. The material properties for Zr-based BMG are: *E*_0_ = 86 Gpa, *v*_0_ = 0.36, *ξ*_0_ = 0.05, *n_D_* = 3, *α* = 0.5, *β* = 0.9 and *σ*_0_ = 125 Mpa. The predictions are compared with the measured results shown in Figure 2, where *ε*_0_ = 0.04 and *m* = 18 are used. All the predictions agree with the experiments very well, and strain-softening effect and the collapse stage can be clearly reflected by the damage model.

**Figure 3:**
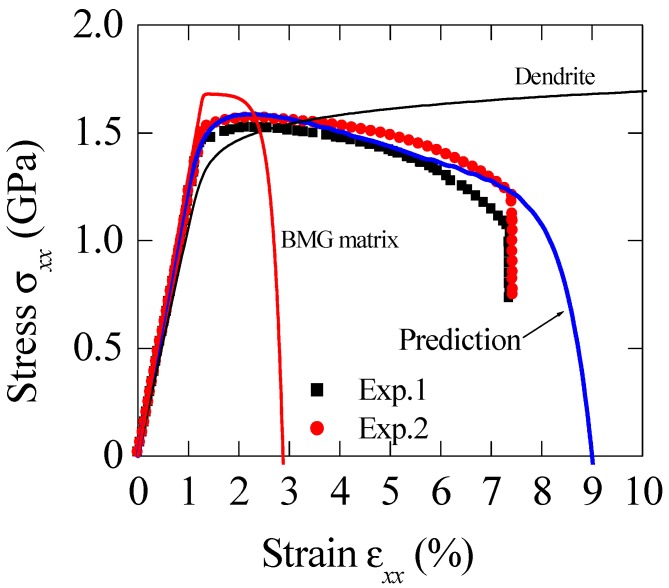
Comparisons of the macroscopic stress–strain relations between the prediction and experiments for BMGCs with particle volume fraction of 43% [19]. *ε*_0_ = 0.1 and *m* = 16 are adopted in the computation.

**Figure 4:**
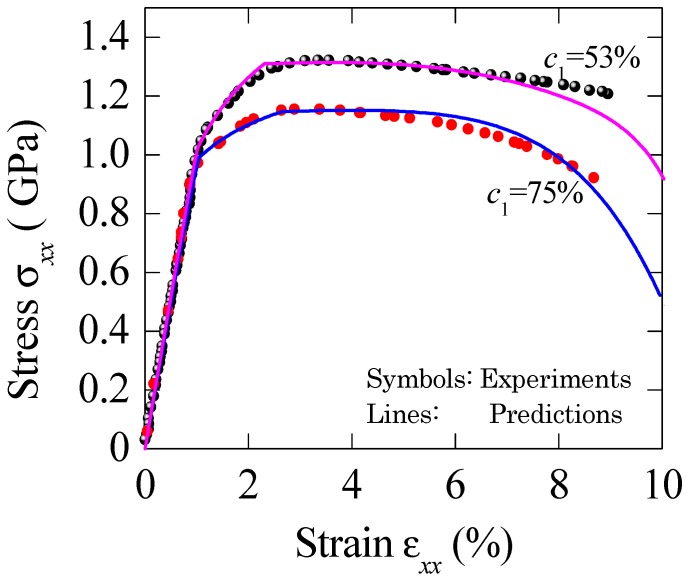
Comparisons of the macroscopic stress–strain relation between the prediction and experiments for BMGCs with different particle concentrations [20]. *ε*_0_ = 0.11 and *m* = 8 are adopted in the computation.

Figure 3 shows the calculated stress–strain relation for BMGC with dendrite concentration of *c*_1_ = 43% [19]. The material properties of constituents are: *E*_1_ = 140.3 Gpa, *ν*_1_ = 0.3, *σ*^1^*_y_*= 1600 Mpa, *n*_1_ = 0.2, *h*_1_ = 388 Mpa, *E*_0_ = 106 Gpa, *ν*_0_ = 0.35, *σ*^0^*_y_*= 1336 Mpa, *n*_0_ = 0.4, *h*_0_ = 688 Mpa, *ε*_0_ = 0.1 and *m* = 16. Figure 4 plots the stress–strain relations for BMG composite with various dendrite volume fractions [20]. Material properties are: *E*_1_ = 127 Gpa, *ν*_1_ = 0.3; *E*_0_ = 90 Gpa, *ν*_0_ = 0.35, *σ*^1^*_y_*= 1 Gpa, *n*_1_ = 0.5, *h*_1_ = 560 Mpa, *σ*^0^*_y_*= 700 Mpa, *n*_0_ = 0.1, *h*_0_ = 740 Mpa, *ε*_0_ = 0.11 and *m* = 8.

BMGC’s deformation increases step by step; a slight work hardening was observed, followed by remarkable improvement in plastic strain level. By including the damage effect, the collapse stage during deformation could be clearly presented. Finally, the predicted results are in good agreement with the measured data. On the other hand, the increase in dendrite loading level will greatly impair the yielding stress. The present comparison confirmed that this model can reflect the dependence of composite strength and ductility on phase volume fractions. 

**Figure 5:**
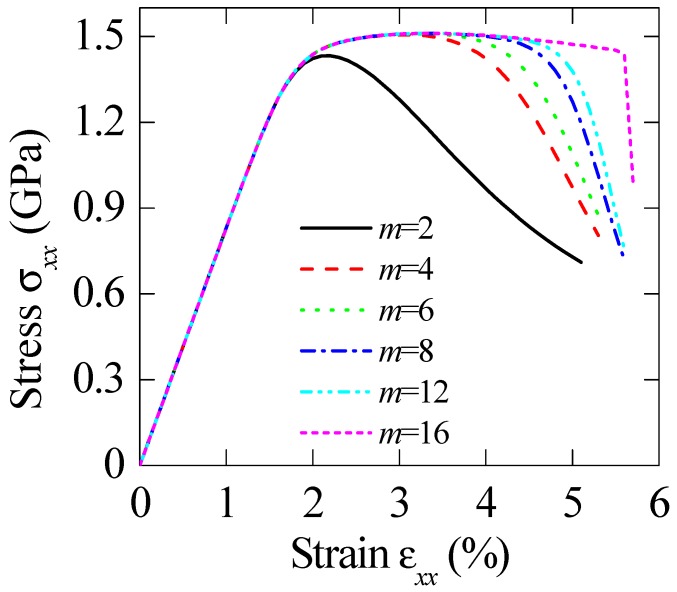
Stress–strain relations of BMGCs with different Weibull modulus *m*, where *ε*_0_ = 0.06 is adopted.

**Figure 6:**
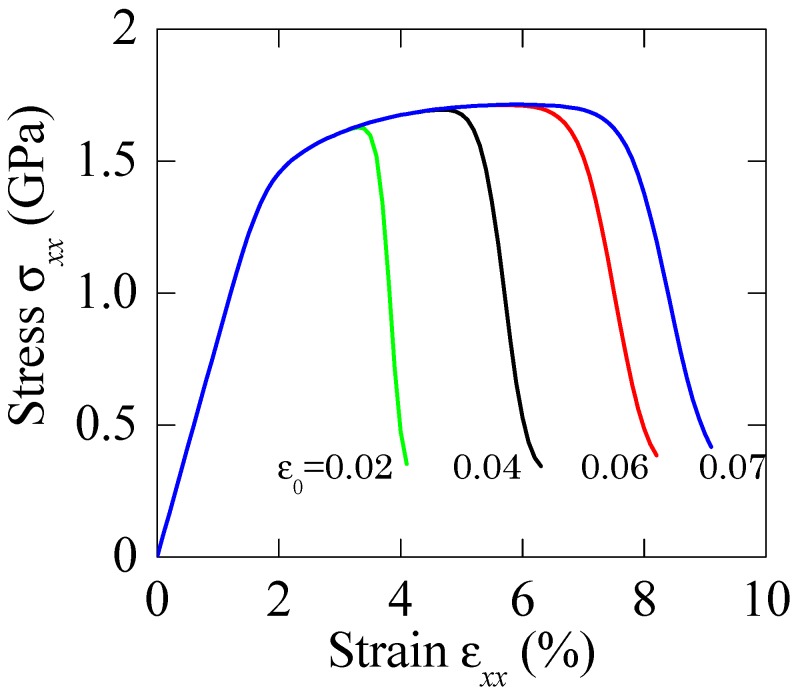
Stress–strain relations of BMGCs with different reference strain *ε*_0_, where *m* = 15 is adopted.

The impacts of model parameters in the present method should be carefully explained. Figure 5 and Figure 6 demonstrate the effect of Weibull modulus *m* and reference strain *ε*_0_ on the overall stress–strain curves, respectively. In Figure 5, the Weibull modulus m ranges from 2 to 16, and the reference strain *ε*_0_ = 0.06. At a given particle volume fraction, the plastic elongation increases with increasing Weibull modulus, which is controlled by the physics meaning of Weibull modulus. Additionally, the dependence of the overall stress–strain relations on reference strain *ε*_0_ is illustrated in Figure 6. As expected, the uniform stretches decrease with the increase in micro-crack density. For the reference strain less than 0.1, the difference in plastic elongation becomes evident.

**Figure 7:**
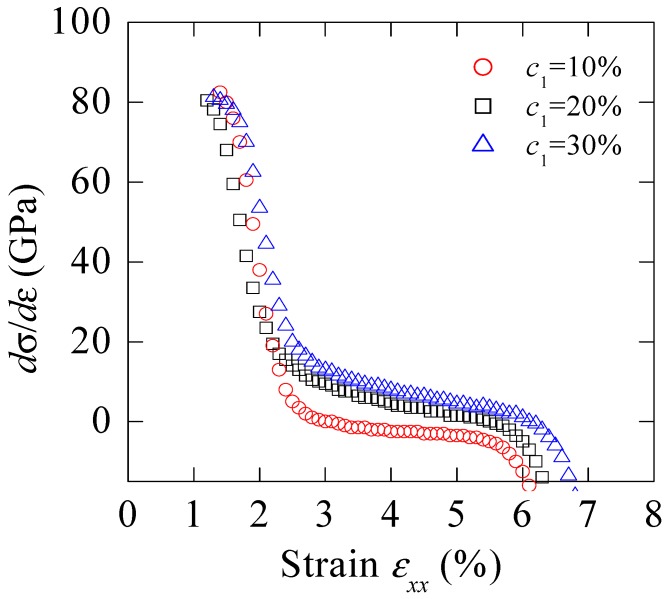
Dependence of work hardening rate *dσ/dε* on particle volume fraction. *ε*_0_ = 0.06 and *m* = 15 are adopted.

Based on the work hardening coefficient, the ability to endure damage can be judged for engineering materials. The reliance of work-hardening rate *dσ*/*dε* on particle concentration is shown in Figure 7. Here, the definition of *dσ*/*dε*, is used to describe the resistance to flow localization, i.e., necking. It is noted that the value of *dσ*/*dε* is higher for BMGCs with higher filler volume fraction. Moreover, the value reduces rapidly with deformation for dilute composites. These curves also show that uniform elongation becomes larger with the higher initial hardening rate. Since the material with a high *dσ*/*dε* can lead to a much uniform plastic flow, which is against the early appearance of deformation localization, and an increased stretch is finally reached.

### 4.2. Discussion

The comparisons between the predictions and experiments indicate that the present model can describe the damage behaviors of BMGCs accurately. Under the multi-axial stress state, the BMG matrix in the composites exhibits a certain degree of plasticity, which is absent for pure BMG under uniaxial loading. Based on the free volume model and iso-hardening flow law for the constituents, the equivalent plastic behaviors of BMG matrix can be determined by the present model.

Based on the Eshelby’s tensor and Mori–Tanaka mean field frame, many micromechanic models have been developed, and are commonly applied under stress-controlled loading. Such models have difficulty describing strain-softening deformation. In the present work, the secant modulus and strain-controlled formula were adopted, and the aforementioned difficulty can be easily avoided. In Rao’s mesoscale model, two important terms need to be performed: The first one is the algorithmic tangent operator, obtained by consistent linearization of the time discretized constitutive equations. The second is a new one and called an affine strain increment. Therefore, their model has very complicated in mathematical formulas. On the other hand, the present model does not need to calculate the tangent stiffness instead of the secant modulus, and is very simple in mathematic form, which is readily realized in programming.

It is expected that particle volume fraction is usually higher than 30%, even exceeding 50% for many BMGCs. The interaction between particles and matrix cannot be well considered by the Mori–Tanaka method. Additionally, shear bands will finally transform into micro-cracks by increasing the applied deformation, and moreover the microstructure evolution is a complicated process, and their effect on the macroscopic performance is not clear yet. These problems should be deeply studied and tackled by improving the present model in the future.

## 5. Conclusions

Based on Weng’s method for dual-phase composites, a meso-mechanical damage model was proposed to depict the tensile failure of BMGCs. Free volume theory was adopted to describe the strain softening of BMG matrix. The strain-based Weibull probability distribution function and percolation theory are used to reflect the damage effect induced by the transformation from shear bands to micro-cracks. The displacement controlled loading was applied to predict the collapse stage during the deformation. The final comparisons with the experiments confirm that the present model can replicate the monotonic tensile stress–strain relations of BMGCs until final failure.
